# Boosting Immunity of the Registered Reports System in Psychology to the Pandemic

**DOI:** 10.3389/frma.2020.607257

**Published:** 2020-11-24

**Authors:** Kyoshiro Sasaki, Yuki Yamada

**Affiliations:** ^1^ Kansai University, Osaka, Japan; ^2^ Kyushu University, Fukuoka, Japan

**Keywords:** registered reports, file drawer problem, idea journals, academic publishing, coronavirus disease 2019, psychology, open science

## Abstract

In psychology, a Registered Reports system is key to preventing questionable research practices. Under this system, manuscripts, including their detailed protocols (i.e., hypothesis, experimental design, sample size, and methods of statistical analysis), are reviewed prior to data collection. If a protocol manuscript is accepted, publication of the full manuscript including the results and discussion is guaranteed in principle regardless of whether the collected data support the registered hypothesis. However, this assurance of publication might be broken under the impact of the COVID-19 pandemic: Begrudging withdrawal of an accepted protocol manuscript due to a difficulty to meet the deadline by compelling reasons (e.g., pandemic) has occurred. The present paper reports the first author’s real-life experience related to the collapse of the assurance of publication in the Registered Reports system and discusses the disbenefits of this collapse. Furthermore, we propose the implementation of a journal section specific to protocol manuscripts as a solution to the crisis of the Registered Reports system.

## Psychology and the Pre-Registration System

The reproducibility of studies in psychology has often been pointed out (e.g., Open Science Collaboration, 2015). The main factor is assumed to be questionable research practices (QRPs: e.g., John et al., 2012; Ikeda et al., 2019). One of the major QRPs is *p*-hacking (e.g., Simmons et al., 2011), which is the practice of seeking out *p*-values convenient for researchers (e.g., adding new data to an analysis until the results support the researchers’ claim). Cherry picking is also a QRP (e.g., Fraser et al., 2018): Reporting only favorable results for researchers and ignoring or hiding unfavorable results. A third QRP is HARKing (Hypothesizing After the Results are Known: Kerr, 1988), in which researchers construct their hypothesis after the results of experiments are known to ensure a good or challenging story. These QRPs inflate the possibility of Type I error, in turn leading to low reproducibility.

A pre-registration system is one way to prevent QRPs (Nosek et al., 2018). In such a system, researchers register the detailed protocol of their studies (e.g., hypothesis, experimental design, sample size, and statistical analysis) on designated websites (e.g., Open Science Framework and AsPredicted) before they begin their experiments. They cannot modify the protocol after registration and in principle, must conduct the experiments and statistical analyses in line with the registration. However, a pre-registration system is also likely to be cracked (Pre-reg hacking: Ikeda et al., 2019; Yamada, 2018). For example, researchers can repeat experiments until they obtain results consistent with the pre-registration (“infinite re-experimenting,” “reset marathon,” or “rerolling”: Yamada, 2018). Researchers can also “pre-”register the protocol after the results of experiments are known (Pre-registering After the Results are Known; PARKing: Yamada, 2018). Moreover, researchers can register multiple similar protocols at numerous registration systems simultaneously and adopt only the suitable pre-registration (Ikeda et al., 2019). These pre-reg hackings (and QRPs) might occur because of the “positive results = win” mode of thinking widespread throughout the science community (Yamada, 2018), whereby a paper with positive or challenging results will be published smoothly. In any case, the pre-registration system has several drawbacks and cannot completely prevent QRPs.

## Registered Reports

Peer-reviewed pre-registration (i.e., the Registered Reports system: e.g., Nosek et al., 2018; Nosek and Lakens, 2014) compensates for shortcomings of the pre-registration system. Under this system, the manuscript including only the detailed protocol is peer-reviewed prior to data collection, and researchers must revise it if reviewers point out flaws. After the manuscript successfully passes this pre-review process (i.e., in-principle acceptance), the protocol manuscript is registered on the pre-registration websites or at each journal as in-principle acceptance (or Stage 1 acceptance), and the full manuscript including the results and discussion sections will essentially be published regardless of whether the collected data support the registered hypothesis. The Registered Reports system decreases the advantages of and motivations for QRPs and pre-reg hackings because publication is guaranteed once the protocol is accepted, and thus no positive or challenging results are necessary. The Registered Reports system can also prevent the publication bias arising when only manuscripts with positive results are published and negative results are never reported (e.g., Mahoney, 1977; Sterling et al., 1995). Moreover, the time of publication is possibly controlled to some extent because the schedule after in-principle acceptance depends mostly on researchers’ activity. Taken together, the Registered Reports system has several merits that mainly stem from an assurance of publication after in-principle acceptance. However, this assurance has been broken under the impact of the COVID-19 pandemic.

## A Case Report of the Assurance of Publication Being Revoked

The first author (KS) and his colleagues submitted a protocol manuscript to a legitimate and trustworthy journal that fortunately had been accepted in principle just before the start of the COVID-19 pandemic (i.e., February 4, 2020, GMT).^1^ In the protocol, they planned to conduct laboratory experiments requiring a relatively large sample size (*N* = 332 in total) and the initial deadline of the full manuscript was two months later from in-principle acceptance (i.e., April 4, 2020, GMT). However, because of the COVID-19 pandemic, they, as well as most researchers, have been rendered unable to conduct any laboratory experiments. Although the action editor kindly extended the deadline for about 5 months (i.e., until September 7, 2020, GMT), the authors cannot expect to be able to start laboratory experiments within this period, and thus they asked the action editor to be allowed to change the protocol from laboratory experiments to online ones; however, they were told that it would be necessary to withdraw and resubmit the protocol manuscript in this case because of a large deviation from the initial plan.^2^ The authors then asked the action editor whether the journal could wait until COVID-19 has been contained for them to submit the full manuscript, stating that they would have been able to complete the planned experiments if they had maintained the original plan (i.e., the laboratory-experiment plan). The action editor and chief editor considered this issue, and then stated that they wished to avoid an open-ended deadline and could only extend the deadline to December 7, 2020 (or January 2021), at the maximum. As COVID-19 appears increasingly unlikely to be contained soon, prolonged or intermittent social distancing is likely to be necessary (Kissler et al., 2020), and thus it would be difficult to meet even the extended deadline. That is, the authors had no choice but to reluctantly withdraw their protocol manuscript, even though the manuscript had originally been accepted in principle and publication of the full manuscript had been promised. As indicated above, there was no fault on the part of the authors and no provision regarding this issue in the submission guidelines, whose contents remain unchanged after the authors received the editorial team’s opinion, just as, so to speak, sane online-game players have been banned although they had played within the rules. Thus, this case indicates that the assurance of publication after in-principle acceptance via the Registered Reports system can collapse due to unpredicted events such as COVID-19.

The case of begrudging withdrawal of an accepted protocol manuscript due to a difficulty to meet the deadline by compelling reasons (e.g., pandemic) should be avoided because researchers in such situations might twist the data to forcibly meet the deadline. This defeats the purpose of the Registered Reports system, which was intended to increase transparency and prevent misconduct and QRPs. Moreover, the withdrawal of an accepted protocol manuscript is tantamount to losing a peer-reviewed article from one’s research history, which would be a terrible blow particularly for Early Career Researchers (ECRs).^3^ Furthermore, no assurance of publication after in-principle acceptance might reduce researchers’ motivation to submit their manuscript to the Registered Reports section, hampering the operation of this system. As this unintended withdrawal of registered reports might occur not only under the impact of the COVID-19 pandemic, solutions are necessary.

## Possible Solutions to the Unwilling Withdrawal of Accepted Protocol Manuscripts

How can we solve the problem of the withdrawal of registered reports? A simple solution would be for journals to flexibly extend the deadline for an indefinite time. However, an indefinite deadline might cause some disbenefits for journals; in the case of the first author, the editorial team clearly stated that an open-ended deadline would not be desirable. Indeed, if a long time passed after in-principle acceptance, it is unclear whether the same editors and reviewers would be available to review the same manuscript again, which would introduce confusion into the publication process. Perhaps, then, an open-ended system should be implemented? For example, a good solution might be for the protocol manuscript to be published by itself upon acceptance and other researchers can perform experiments in line with the protocol and publish manuscripts consisting largely of the results and discussion. This idea is based on the notion of a division of labor between the pre-registration and experimental groups in the Registered Reports system (Yamada, 2018; Ikeda et al., 2019; Yamada, 2020). In particular, because most researchers cannot perform laboratory experiments during the COVID-19 pandemic but will still have many interesting ideas, there might be a great demand for a journal section dedicated to protocol manuscripts. Moreover, a previous study suggests that a specific journal section for protocol manuscripts is easily realizable through micropublishing (Yamada, 2020). If a journal section specific to protocol manuscripts is implemented, the problem of the withdrawal of registered reports will disappear.

A journal section specific to protocol manuscripts would appear to offer benefits after the end of the COVID-19 era. It is possible that if hypothesis builders and experimenters are different, pressures for QRPs will be largely eliminated (Yamada, 2020). Moreover, many research groups are likely to conduct the same experiments simultaneously, which will ease and speed up the confirmation of the robustness of effects; this is similar to multi-lab replication (e.g., Klein et al., 2014; Ebersole et al., 2016; Klein et al., 2018). Additionally, although at this time only all-rounders (i.e., those who can build interesting hypotheses and have the skills to perform the experiments and complicated analyses) can come under the spotlight in psychology, the establishment of flexible research structures based on the division of labor will make it easier for different types of researchers to flourish in academia (Yamada, 2019). Briefly, the creation of journal sections dedicated to protocol manuscripts and the resultant division of labor are keys to resolving current problems in the psychology community.

## Conclusion

The Registered Reports system is highly beneficial to psychological science by promoting transparency and reproducibility. Assurance of publication after in-principle acceptance is central to the Registered Reports system. Therefore, the collapse of this assurance means the death of this system. A journal section dedicated to protocol manuscripts would help resolve the crisis in registered reports. This proposal should make the Registered Reports system more flexible and thus, the system possibly comes to function properly under various kinds of unexpected situations (eg, pandemic). Last but not least, thanks to the valuable efforts of the editors of both journals, the in-principle acceptance of our protocol manuscript has been transferred to another journal (Chambers, 2020; Sasaki et al., 2020). This should be the first case of a cross-journal transfer of the registered reports. With this case as a start, the Registered Reports system might develop into the one detached and free from journals.

## Author Contributions

KS: Conceptualization, Funding Acquisition, Project Administration, Resources, Writing—Original Draft, and Writing—Review and Editing. YY: Conceptualization, Funding Acquisition, Supervision, Resources, Writing—Original Draft, and Writing—Review and Editing.

## Funding

The present study was supported by JSPS KAKENHI (JP19K14482 to KS, and JP16H03079, JP17H00875, JP18K12015, and JP20H04581 to YY).

## Conflict of Interest

The authors declare that the research was conducted in the absence of any commercial or financial relationships that could be construed as a potential conflict of interest.

## Footnotes

1 We have no intention of criticizing a specific journal. This opinion piece is provided in an effort to improve the Registered Reports system based on our experience on this occasion.

2 In several studies of our authors’ groups, changes of protocol from laboratory to online experiments were admitted. Approval of protocol changes from laboratory to online experiments might depend on the purpose and methods of the studies.

3 The first author obtained his Ph.D. 4 years ago and thus is also an ECR.
